# Serum GP73 - An Additional Biochemical Marker for Liver Inflammation in Chronic HBV Infected Patients with Normal or Slightly Raised ALT

**DOI:** 10.1038/s41598-018-36480-3

**Published:** 2019-02-04

**Authors:** Meijuan Wei, Zhengju Xu, Xingnan Pan, Xiaoman Zhang, LiGuan Liu, Bishuang Yang, Yuxia Chen

**Affiliations:** 1 Clinical Liver Center, The 910th Hospital of the People’s Liberation Army, Quanzhou, 362000 China; 2 Clinical Liver Center, Decheng hospital of Quanzhou/Affiliated of Huaqiao University, Quanzhou, 362000 China

## Abstract

This study aimed to assess the feasibility of GP73 as a diagnostic marker for liver inflammation and fibrosis in chronic HBV patients with normal or slightly raised ALT (<2 ULN) and to develop models based on GP73 and other biochemical parameters to improve diagnostic accuracy. Serum GP73 levels were analyzed in 220 chronic HBV patients with normal or slightly raised ALT who underwent liver biopsy. The results showed that the area under the receiver operating characteristic (ROC) curve (AUC) was 0.806 for predicting significant liver inflammation (≥G2), while it was 0.742 for predicting significant fibrosis (≥S2). These results suggest that GP73 has higher diagnostic value for liver inflammation than liver fibrosis. Combining GP73, AST and ALB, as a diagnostic model for predicting significant liver inflammation, resulted in superior diagnostic performance over GP73 alone (AUC value increased from 0.806 to 0.854, z = 2.299, P = 0.021). By applying this diagnostic model, over 80% of chronic HBV patients with normal or slightly raised ALT will be correctly identified and hence avoid delay in diagnosis and treatment. In conclusion, GP73 would be an additional serum marker for predicting liver inflammation and fibrosis in chronic HBV patients with normal or slightly raised ALT.

## Introduction

Hepatitis B virus (HBV) is one of the most common infectious diseases in the world with more than 240 million people infected with HBV worldwide. Infection with HBV can be either acute or chronic. In adults, HBV infections have a relatively low rate of chronicity (around 5%), while it results in a high persistence rate in neonates^1,2^. Although, most chronic infections are asymptomatic, HBV carriers are still at risk of hepatic inflammation and fibrosis, which can lead to advanced liver diseases including cirrhosis and hepatocellular carcinoma^3^. Therefore, close follow up and monitoring is recommended for HBV carriers to avoid disease progression. Accordingly, it is highly recommended that HBV carriers with moderate or severe chronic hepatitis, indicated by abnormal levels of alanine-aminotransferase (ALT, ULN < 40 U/L), should receive antiviral therapy^4^. In this context, it is important to note that although the serum level of ALT is the most commonly used parameter for assessing hepatitis activity, it has been reported that 24.6% to 61.9% of chronic HBV infected patients with either normal or slightly raised ALT (<2 ULN) were diagnosed with moderate or severe hepatic inflammation and/or fibrosis^5–7^. Therefore, it is quite possible that patients with ongoing liver injury might not be identified due to having normal levels of ALT, and therefore not receiving the benefit of antiviral therapy. On the other hand, liver biopsy is the current “gold standard” for evaluating hepatic pathology. However, the procedure is limited by its invasive nature and the risk of complications^8,9^. Therefore, identification of new surrogate biomarkers with high diagnostic and prognostic efficiencies for liver injury is urgently needed.

Golgi Protein-73 (GP73), a novel type II Golgilocalized integral membrane protein also known as GOLPH2 or GOLM1, is predominantly expressed in epithelial cells of many human tissues^10^. Serum GP73 levels were found to be significantly raised in patients with a variety of acute or chronic liver diseases^11,12^, and especially in patients with hepatocellular carcinoma^13^. In our previous studies, we found that changes in serum GP73 levels were closely associated with changes in the degree of liver injury since serum GP73 levels were positively correlated with liver pathological grading and staging in patients with chronic HBV infection^14,15^. Whether GP73 could be used for liver inflammation and fibrosis in chronic HBV infected patients with normal or slightly raised ALT has not been reported.

In an attempt to identify a non-invasive biomarker for diagnosis of liver inflammation and fibrosis in HBV patients with normal or slightly raised ALT, we investigated the diagnostic performance of serum GP73. Ultimately, we proposed two diagnostic models consisting of combined biochemical parameters to improve the diagnostic accuracy of liver inflammation and fibrosis.

## Results

### Patient Characteristics

A total of 220 patients with chronic HBV infection and normal or slightly raised ALT (<2 ULN) were enrolled. The demographic and clinical characteristics of the enrolled patients at the time of liver biopsy were listed in Table 1. The majority of the patients (64.6%) had no-activity or minimal (G0-1) liver inflammation, while only 2% had severe inflammation grade (G4). Additionally, nearly half of the patients (48%) had stageS0-1 fibrosis.

**Table 1:** Demographic and clinical characteristics of all participants.

Variable	Patients’ Characteristics Estimation Group (n = 220)
Age, years	35 (29–43)
Sex, male, (%)	155 (70.45)
GP73, ng/ml	51.34 (37.47–75.33)
ALB, g/L	42.90 (40.33–45.70)
TBIL, μmol/L	15.85 (12.10–21.08)
ALT, IU/L	46.75 (31.93–60.98)
40, n (%)	86 (39.09)
40–80, n (%)	134 (60.91)
AST, IU/L	30.70 (25.00–38.25)
GGT, IU/L	30.85 (19.93–49.25)
ALP, IU/L	76.20 (61.90–93.30)
CHE, IU/L	7929.00 (6772.00–9427.00)
PLT, (10^9^/L)	193.5 (158.30–228.50)
HBV DNA, (log10 copies/mL)	6.94 (5.37–7.73)
3.3, n (%)	1 (0.45)
3.3~4, n (%)	7 (3.18)
4~7, n (%)	109 (49.55)
7, n (%)	103 (46.82)
HBeAg positive, n (%)	145 (65.9)
HBeAg negative, n (%)	75 (34.1)
Anti-HBe positive, n (%)	67 (30.45)
HBsAg levels, (log10 IU/mL)	3.71 (3.26–4.19)
**grade of inflammation, n (%)**	
G0–1	142 (64.55%)
G2	53 (24.09%)
G3	20 (9.1%)
G4	5 (2.27%)
**Stage of fibrosis n, (%)**	
S0-1	106 (48.18%)
S2	41 (18.64%)
S3	26 (11.82%)
S4	47 (21.36%)

ALB, albumin; TBIL, total bilirubin; ALT, alanine aminotransferase; AST, aspartate aminotransferase; GGT, γ-glutamyltransferase, ALP, alkaline phosphatase; CHE, cholinesterase; PLT, Platelet counts; HBV DNA, hepatitis B viral DNA; HBeAg, hepatitis B virus e antigen; Anti-HBe, hepatitis B virus e antibody; HBsAg, hepatitis B virus surface antigen; G hepatitis B virus e antigen G, hepatic necroinflammation activity grade; S, liver fibrosis stage.

### Univariate and Multivariate Analysis

Based on inflammation grade, all patients were divided to two groups: 1) patients with no significant inflammation (<G2); and 2) patients with significant inflammation (≥G2). Demographic and biochemical parameters were compared between both groups (Table 2).While all variables, except age, sex, TBIL, ALP, HBV DNA, HBeAg and Anti-HBe, were significantly different between the two groups by univariate analysis, only GP73, ALB and AST were identified by multivariable analysis as independent factors significantly associated with the grade of liver inflammation (Table 2). Similarly, based on fibrosis stage, patients were divided into two groups: 1) patients with no significant fibrosis (<S2); and 2) patients with significant fibrosis (≥S2). When demographic and biochemical parameters were compared between the both groups (<S2 vs. ≥S2), all variables except sex, ALB, ALT, ALP, HBV DNA, HBeAg and Anti-HBe, were significantly different between the two groups by univariate analysis (Table 3). Subsequently, multivariate analysis identified GP73 and PLT count as independent factors significantly associated with the stage of liver fibrosis (Table 3).

**Table 2:** Univariate and multivariate analysis between patients with or without significant inflammation.

Variable	No Significant Inflammation (n = 142)	Significant Inflammation (n = 78)	Univariate	Multivariate	
			*P* Value	OR (95%CI)	*P* Value
Age, years	35 (29-40.25)	35.5 (30–45)	0.253	0.993 (0.956–1.031)	0.702
Sex, male, (%)	110 (77.5)	52 (66.67)	0.082		
GP73, ng/ml	44.53 (33.03–58.60)	76.85 (52.60–113.30)	0.001	1.043 (1.027-1.06)	0.0001
ALB, g/L	43.55 (42.00–46.53)	41.25 (38.40–43.90)	0.001	0.834 (0.746–0.932)	0.001
TBIL, μmol/L	15.20 (11.88–20.53)	16.70 (12.49–21.70)	0.206	1.016 (0.9629–1.072)	0.567
ALT, IU/L	42.50 (29.85–59.00)	52.55 (40.88–63.48)	0.003	1.005 (0.979–1.031)	0.728
AST, IU/L	28.00 (23.15–34.48)	35.30 (30.08–47.45)	0.001	1.055 (1.014–1.097)	0.007
GGT, IU/L	26.00 (17.90–43.08)	38.65 (27.40–60.68)	0.001	0.999 (0.988–1.01)	0.885
ALP, IU/L	75.50 (62.78–90.00)	77.50 (60.03–103.4)	0.435	0.996 (0.979–1.013)	0.651
CHE, IU/L	8152 (7143–9494)	7436 (6269–8777)	0.005	1.000 (0.999–1.0001)	0.953
PLT, (10^9^/L)	201.00 (169.50–236.50)	177.5 (140.5–218.0)	0.004	0.994 (0.985–1.002)	0.137
HBV DNA, (log10 copies/mL)	6.81 (5.40–7.61)	7.17 (5.30–7.89)	0.299	1.454 (1.09–1.938)	0.131
HBsAg levels, (log10 IU/mL)	3.67 (3.43–4.11)	3.79 (3.16–4.24)	0.071	1.173 (1.089–1.350)	0.097
HBeAg positive, n (%)	91 (64.08)	54 (69.23)	0.461		
Anti-HBe positive, n (%)	46 (32.39)	21 (26.92)	0.446		

ALB, albumin; TBIL, total bilirubin; ALT, alanine aminotransferase; AST, aspartate aminotransferase; GGT, γ-glutamyltransferase, ALP, alkaline phosphatase; CHE, cholinesterase; PLT, Platelet counts; HBV DNA, hepatitis B viral DNA; HBeAg, hepatitis B virus e antigen; Anti-HBe, hepatitis B virus e antibody; HBsAg, hepatitis B virus surface antigen.

**Table 3:** Univariate and multivariate analysis between patients with or without significant fibrosis.

Variable	No Significant Fibrosis (n = 106)	Significant Fibrosis (n = 114)	Univariate	Multivariate	
			*P* Value	OR (95%CI)	*P* Value
Age, years	33 (27–39)	37.5 (32–44)	0.002	1.02 (0.985–1.056)	0.267
Sex, male, (%)	80 (75.5)	88 (77.2)	0.764		
GP73, ng/ml	43.23 (31.51–57.57)	62.69 (93.35–46.55)	0.001	1.034 (1.019–1.05)	0.0001
ALB, g/L	43.30 (41.65–45.43)	42.25 (39.15–45.85)	0.085	0.978 (0.891–1.074)	0.642
TBIL, μmol/L	14.65 (11.20–19.88)	16.55 (13.05–22.60)	0.024	1.018 (0.972–1.067)	0.454
ALT, IU/L	45.15 (29.70–60.63)	48.20 (32.65–61.20)	0.116	0.999 (0.977–1.022)	0.959
AST, IU/L	28.10 (22.55–35.15)	33.15 (26.09–44.48)	0.001	1.013 (0.98–1.046)	0.458
GGT, IU/L	24.00 (17.25–38.85)	38.80 (24.08–57.98)	0.001	1.002 (0.993–1.01)	0.728
ALP, IU/L	74.00 (59.80–90.18)	78.15 (65.20–95.85)	0.072	1.008 (0.993–1.024)	0.288
CHE, IU/L	8337 (7446–9695)	7417 (6319–8966)	0.001	0.999 (0.999–1.0000)	0.152
PLT, (10^9^/L)	210.5 (180.00–243.3)	174.0 (139.8–213.0)	0.001	0.989 (0.982–0.996)	0.002
HBV DNA, (log10 copies/mL)	7.01 (5.79–7.65)	6.67 (5.05–7.78)	0.122	1.061 (0.921–1.938)	0.087
HBeAg positive, n (%)	63 (59.43)	79 (69.30)	0.158		
Anti-HBe positive, n (%)	38 (35.85)	32 (28.07)	0.246		

ALB, albumin; TBIL, total bilirubin; ALT, alanine aminotransferase; AST, aspartate aminotransferase; GGT, γ-glutamyltransferase, ALP, alkaline phosphatase; CHE, cholinesterase; PLT, Platelet counts; HBV DNA, hepatitis B viral DNA; HBeAg, hepatitis B virus e antigen; Anti-HBe, hepatitis B virus e antibody.

### Estimation of the Models

Next, we developed two score systems using the logarithmic values (Ln) of significant independent variables and their coefficients for predicting inflammation or fibrosis based on the following binary logistic regression: Model1 (Inflammation Probability) = 14.66 + 2.4 Ln(GP73) + 1.99 Ln(AST) − 7.15 Ln(ALB) (Table 4); and Model2 (Fibrosis Probability) = 9.42 + 1.89 Ln(GP73) − 2.63 Ln(PLT) (Table 5).

**Table 4:** Logistic regression analysis for the factors associated with inflammation.

Variable	B	SE	Wald	P value	OR (95%CI)
Ln (GP73)	2.40	0.462	26.863	0.001	10.937 (4.426–27.026)
Ln (ALB)	−7.15	2.161	10.940	0.001	0.001 (0–0.054)
Ln (AST)	1.99	0.596	11.103	0.001	7.277 (2.264–23.385)
Constant	14.66				

ALB, albumin; ALT, alanine aminotransferase; B, regression coefficient; S.E., standard error; OR, odds ratio; CI, confidence interval; Ln, natural logarithm.

**Table 5:** Logistic regression analysis for the factors associated with fibrosis.

Variable	B	SE	Wald	P value	OR (95%CI)
Ln (GP73)	1.89	0.359	27.814	0.001	6.646 (3.288–13.436)
Ln (PLT)	−2.63	0.649	16.452	0.001	0.072 (0.02–0.256)
Constant	9.42				

PLT, Platelet counts; B, regression coefficient; S.E., standard error; OR, odds ratio; CI, confidence interval; Ln, natural logarithm.

### Comparison of Diagnostic Efficacy

**Figure 1:**
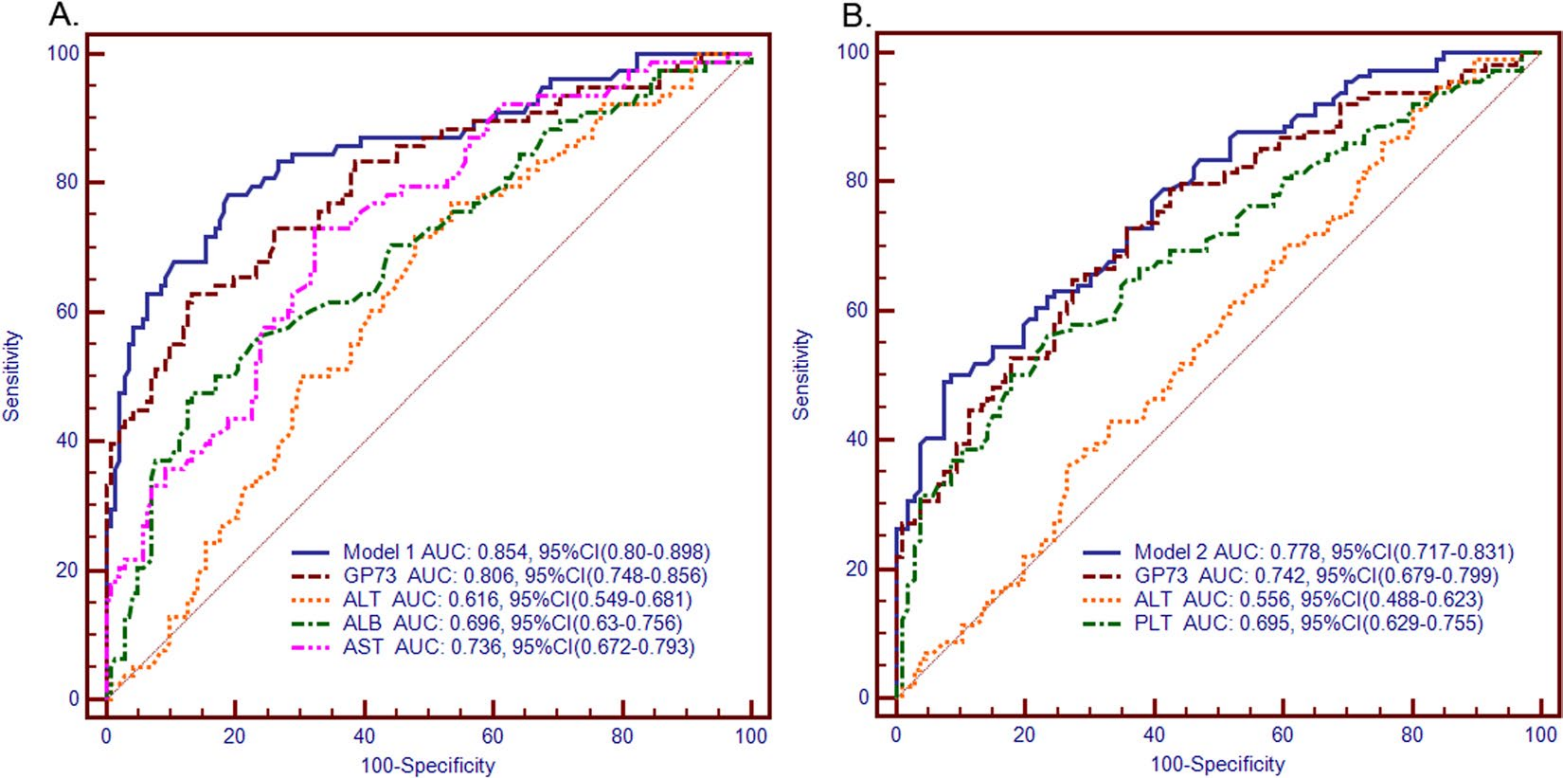
Comparison of Diagnostic Efficacy in Chronic HBV Infected Patients with Normal or Slightly Raised ALT. (**A**) ROC analysis of GP73, ALB, AST, ALT and Model1 for significant liver inflammation; (**B**) ROC analysis of GP73, PLT, ALT and Model2 for significant liver fibrosis.

To estimate the optimal cut off value of GP73 for predicting significant liver inflammation (≥G2) or significant liver fibrosis (≥S2), the ROC curve analysis was used. This analysis identified 85.7 ng/ml as an optimal cut off value of GP73 to distinguish between patients with and without significant liver inflammation among HBV patients with normal of slightly raised ALT levels. A GP73 cut off value of 85.7 ng/ml resulted in an AUC of 0.806 which was significantly higher than that of ALT (0.616) (z = 3.917, P < 0.001), and the sensitivity and specificity were 43.59% and 97.18% respectively (Fig. 1A and Table 6). Interestingly, using 85.7 ng/ml as a cut off for GP73, 34 (43.59%) of the 78 patients with significant inflammation by liver biopsy were correctly identified, while only four (2.82%) of the 142 patients with serum GP73 level higher than 85.7 ng/ml had no significant inflammation by liver biopsy. When significant liver fibrosis was considered, the ROC analysis identified 84.49 ng/ml as an optimal cut off value of GP73 to distinguish between patients with and without significant liver fibrosis. The GP73 cut off value of 84.49 ng/ml resulted in an AUC of 0.742, which also significantly higher than that of ALT (0.556), and the sensitivity and specificity were 30.70% and 96.23% respectively (Fig. 1B and Table 7). Using 84.49 ng/ml as a cut off value for GP73, significant fibrosis could be predicted in only 35 (30.7%) of 114 patients with significant fibrosis determined by liver biopsy, reflecting the low sensitivity of this cut off value.

**Table 6:** Diagnostic values of GP73, ALB, AST, ALT and Model1 for predicting significantly inflammation.

Variable	AUC (95%CI)	PPV (%)	NPV (%)	Sens (%)	Spec (%)	DA (%)	YI
GP73	0.806 (0.748–0.856)	89.5	75.8	43.59	97.18	78.2	0.494
ALB	0.696 (0.63–0.756)	72.5	72.8	37.18	92.25	72.3	0.341
AST	0.736 (0.672–0.793)	72.2	71.7	33.33	92.96	71.8	0.407
ALT	0.616 (0.549–0.681)	66.7	65.0	2.56	99.30	65.0	0.239
Model1	0.854 (0.80–0.898)	84.5	82.1	62.82	93.66	82.7	0.592

ALB, albumin; ALT, alanine aminotransferase; AST, aspartate aminotransferase; AUC, the area under the receiver operating characteristic curve; CI, confidence interval; PPV, positivepredictive value; NPV, negative predictive value; Sens, sensitivity; Spec, specificity; DA, accuracy; YI, Youden index.

**Table 7:** Diagnostic values of GP73, PLT, ALT and Model2 for predicting significantly fibrosis.

Variable	AUC (95%CI)	PPV (%)	NPV (%)	Sens (%)	Spec (%)	DA (%)	YI
GP73	0.742 (0.679–0.799)	89.74	56.35	30.70	96.23	62.27	0.376
PLT	0.695 (0.629–0.755)	71.9	61.8	56.14	72.42	65.91	0.326
ALT	0.556 (0.488–0.623)	54.9	72.0	93.86	16.98	44.09	0.110
Model2	0.778 (0.717–0.831)	86.4	63.0	50.00	91.51	69.55	0.4158

PLT, Platelet counts; ALT, alanine aminotransferase; AUC, the area under the receiver operating characteristic curve; CI, confidence interval; PPV, positivepredictive value; NPV, negative predictive value; Sens, sensitivity; Spec, specificity; DA, accuracy; YI, Youden index.

When ROC analysis was performed on Model1 consisting of combined GP73, ALB and AST for prediction of significant liver inflammation, a cut off value of 5.31 was identified. This cut off value produced an AUC of 0.854 (Fig. 1A and Table 6), which was significantly higher than that of GP73 (0.806) alone (z = 2.299, P = 0.021) for prediction of significant liver inflammation. Sensitivity, specificity, PPV, NPV, and diagnosing accuracy of the cut off value of 5.31 for Model1 were 62.82%, 93.66%, 84.5%, 82.1% and 82.7%, respectively. When the cutoff value was set at 4.18, the sensitivity, specificity, PPV and NPV were 82.05%, 75.35%, 64.64% and 81.06%, respectively. Accordingly, 64 (82.05%) of the 78 patients with significant inflammation by liver biopsy were correctly identified. When Model2 consisting of combined GP73 and PLT for prediction of the stage of liver fibrosis was considered, ROC analysis identified 3.7 is an optimal cut off value. This cut off value produced an AUC of 0.778 (Fig. 1B and Table 7), which was higher than that of GP73 (0.742) alone, however the difference was not significant (z = 1.71, P = 0.087). Sensitivity, specificity, PPV, NPV and diagnosing accuracy of the cut off value of 3.7 for Model2 were 50%, 91.51%, 86.4%, 63% and 69.55% respectively.

## Discussion

A critical decision for chronic HBV infected patients is when to initiate antiviral therapy. Usually, the antiviral therapy is initiated in patients showing at least moderate hepatocyte injury (G2) and notably raised ALT levels (>2 ULN)^16^. However, the level of serum ALT may not reflect the correct nature of the hepatic pathology^17^. If strict adherence to practice guidelines of chronic hepatitis B is applied, some chronic HBV infected patients with normal or slightly raised ALT (<2 ULN), but with hepatic inflammation and/or fibrosis might not be identified and be deprived the benefit of antiviral treatment. In several clinical settings, GP73 was shown to be upregulated in a variety of liver diseases, including alcohol-induced liver disease, virus-induced hepatitis (e.g., hepatitis B and C), autoimmune hepatitis, liver cirrhosis, and hepatocellular carcinoma cells (HCC)^18–20^. It was reported that serum GP73 levels were correlated with fibrosis stage and would be a robust biomarker for diagnosis of significant fibrosis in patients with chronic HBV infection^21,22^. However, in our previous studies, we found that the correlation between GP73 and liver inflammation was stronger than that of liver fibrosis^15^. Interestingly, in the present study, serum GP73 was able to predict significant liver inflammation and fibrosis in chronic HBV infected patients with normal or slightly raised ALT. Serum GP73 level demonstrated a high specificity (97.18%) for predicting liver inflammation when the cutoff value was set at 85.7 ng/ml, and the rate of misdiagnosis was only 2.82%. For predicting liver fibrosis, serum GP73 level also demonstrated a high specificity (96.23%) for predicting liver fibrosis when the cutoff value was set at 84.49 ng/ml. Notably, our current results showed that the sensitivity, NPV and diagnosing accuracy of GP73 for predicting liver inflammation was higher than those for predicting liver fibrosis. Moreover, the AUC of GP73 for predicting liver inflammation was higher than for predicting fibrosis (0.806 vs 0.742). Therefore, these results support our previous findings that serum GP73 level is more closely related to hepatic inflammation than to liver fibrosis. What cause the elevation of serum GP73 in liver diseases, hepatocellular injury or fibrosis is still unknown. It was reported that the primary triggers of GP73 expression was the progressive tissue remodeling and fibrogenesis in chronic liver disease^19^. It was also reported that the expression of GP73 could be induced by the proinflammatory cytokines IL-6^23^ and interferon gamma (IFN-γ)^18^ *in vitro*, with serum GP73 levels possibly upregulated under inflammatory conditions^12,24^ *in vivo*. Our findings supported the statement that the elevation of serum GP73 level was triggered by liver inflammatory injury. Since hepatic fibrosisis a common consequence of liver inflammation, we hypothesized that the increased serum GP73 levels in patients with hepatic fibrosis were also attributed to hepatocellular injury. When liver injury occurs, the hepatic macrophages (Kupffer cells) produce a variety of growth and immunomodulating mediators including inflammatory cytokines, especially TNF-α, which has been linked to the pathogenesis of hepatocyte apoptosis and hepatic injury^25–27^. Subsequently, the expression of GP73 in hepatocytes is upregulated by inflammatory cytokines and then released into the extracellular space via proprotein convertase (PC) mediated cleavage, which results in increased GP73 levels in the blood^28^. Furthermore, in our previous studies, we also found that there were significant positive correlations between serum GP73 levels and the expression levels of TNF-α, IL-2 and IL-12 in chronic HBV infected patients with complete response to IFN treatment^29^. This supports the link between GP73 and inflammation.

To improve the diagnostic accuracy of liver inflammation or fibrosis in HBV patients with normal or slightly raised ALT level, biomathematical models were established using a combination of factors identified as independent predictors in multivariate analysis. Model1 consisted of a combination of GP73, ALB and AST, which significantly improved the detection of liver inflammation in patients with normal or slightly raised serum ALT, compared to GP73 alone (AUC value increased from 0.806 to 0.854, p < 0.05). The sensitivity of Model1 was increased to 62.82% compared to GP73 (43.59%), while maintaining a high specificity (93.66%). When the cutoff value was set at 4.18, the sensitivity was further increased to 82.05%, although with a relatively low specificity (75.35%). This indicates that over 80% of chronic HBV infected patients with normal or slightly raised ALT could avoid delays in diagnosis and treatment. Model2 consisted of a combination of GP73 and PLT for prediction of significant fibrosis. This model also improved diagnostic performance, however, this improvement was not significantly different from the diagnostic performance of GP73 alone.

In conclusion, GP73 could be used as an additional serum marker for the diagnoses of liver inflammation and fibrosis in chronic HBV infected patients with normal or slightly raised ALT. Moreover, the diagnostic performance of GP73 for liver inflammation is stronger than for liver fibrosis. The combination of serum levels of GP73, AST and ALB establish a diagnostic model that significantly improved the diagnostic performance for liver inflammation in patients with normal or slightly raised serum ALT. By applying Model1, over 80% of chronic HBV infected patients with normal or slightly raised ALT could be correctly identified and avoid delay in diagnosis and treatment.

## Patients and Methods

### Patients

This retrospective study was carried out in accordance with the institutional ethical guidelines which was approved by the Ethics Committee of the 910 Military hospital, Quanzhou, China. Informed consents were collected from all patients enrolled in this study. Patients with chronic HBV infection who underwent liver biopsy in the Clinical Liver Disease Center (180 Military hospital) between January 2012 and November 2016 were recruited, and medical records were reviewed retrospectively. All patients were selected corresponding to the following criteria: (1) HBsAg and HBV DNA positivity for more than 6 months; (2) normal or slightly raised serum ALT (<2 ULN, ULN < 40 U/L); (3) absence of co-infection with hepatitis C or hepatitis D; (4) absence of HIV, pregnancy, diabetes, thyroid disease, tuberculosis, autoimmune liver diseases and serious cardiac, lung, kidney, nerve and mental diseases, haemochromatosis, or Wilson disease; (5) absence of concomitant drug or alcohol related chronic liver disease; and (6) absence of fatty liver or non-alcoholic steatohepatitis.

### Histologic Grading and Staging

After collecting patient written informed consent, ultra-sonographic-guided liver biopsy was performed using 16G disposable needles (C. R. Bard, Inc., Murray Hill, NJ, USA) according to a standardized protocol. A minimum of 15 mm of liver tissue with at least six portal tracts was required for diagnosis. Specimens were fixed, paraffin-embedded, and stained with hematoxylin-eosin, reticular fiber stain and Masson’s trichrome stain, and examined under a light microscope (Olympus, BX51). The liver biopsies were evaluated independently in a blinded fashion by the same pathologist as for the clinical data. Histological grading of inflammation and staging of liver fibrosis were determined according to Scheuer’s classification^30^: G0 = no activity, G1 = minimal activity, G2 = mild activity, G3 = moderate activity, and G4 = severe activity; S0 = no fibrosis, S1 = expansion of portal fibrotic area, S2 = fibrosis observed in and around portal area, may have formed fibrous septum, S3 = clear fibrosis and hepatic lobule structure disorder but no cirrhosis, and S4 = early cirrhosis.

### Biochemical Measurements and Detection of Serum GP73 Levels

Demographic data were recorded for all patients. Biochemical and hematologic investigations were performed during the week before liver biopsy. Patients’ biochemical parameters including ALT, total bilirubin(TBIL), albumin(ALB), aspartate aminotransferase(AST), γ-glutamyltransferase(GGT), alkaline phosphatase(ALP), and cholinesterase(CHE) were detected using standard hospital-based laboratory procedures on a ROCHE Modular P800 Automatic Biochemical Analyzer (Roche Diagnostics, Mannheim, Germany). Platelet counts(PLT) were detected by an automatic blood cell analyzer (Sysmex XN-3000, Japan). Quantitative enzyme-linked immunosorbent assay (ELISA) kits (Hotgen Biotech Co. Ltd., Beijing, China) were used to detect the serum GP73 levels according to manufacturer’s instructions. Hepatitis B viral DNA (HBV DNA) levels were determined by quantitative PCR (Shenyou Biotec, Shanghai, China) using the Mx3000P System (Agilent Technologies, Santa Clara, CA, USA). Hepatitis B virus surface antigen (HBsAg), Hepatitis B virus e antigen (HBeAg) and hepatitis B virus e antibody (Anti-HBe) levels were determined by electrochemiluminescence immunoassay (Elecsys 2010; Roche Diagnostics, Indianapolis, IN, USA).

### Statistical Analysis

Statistical analyses of the data were performed using SPSS 19.0 (SPSS, Chicago, IL, USA) and STATA 11.0 programs. Continuous variables were expressed as the median (centile 25; centile 75). Kolmogorov-Smirnov tests were used to assess for normality of the data. For continuous variables, the differences were assessed using independent samples t-tests in normal distribution data and Mann-Whitney U-test in abnormal distribution data. For categorical variables, the differences were assessed using Chi-square tests. Then, all variables were included in univariate and multivariate forward stepwise logistic regression analysis to determine the independent predictors of the absence or presence of significant inflammation or fibrosis. Mathematical models (inclusion criteria: P < 0.05, exclusion criteria: P > 0.05) were established by modeling the values of the independent predictors and their coefficients of regression. For each of the models, the diagnostic values were assessed using the area under the receiver operating characteristic (ROC) curves. Areas under the ROC (AUC) of different diagnostic methods were compared using the method of Hardey. Detailed diagnostic values were evaluated by sensitivity, specificity, positivepredictive value (PPV), negative predictive value (NPV), Youden index and accuracy.
